# Toward Success While Tackling the Change in A Pandemic Age: Path-Goal Theory Leadership as a Win-Win Gadget

**DOI:** 10.3389/fpsyg.2022.944145

**Published:** 2022-07-06

**Authors:** Philip Saagyum Dare, Atif Saleem

**Affiliations:** ^1^Faculty of Education, Monash University, Melbourne, VIC, Australia; ^2^College of Teacher Education, Zhejiang Normal University, Jinhua, China

**Keywords:** educational leadership, path-goal theory, leadership styles, school leaders, COVID-19, navigated environment, epidemics, managing change

## Introduction

COVID-19 has drastically altered leadership beliefs and discourse. Despite the fact that school leaders continue to lead their institutions, they do so under circumstances they never envisioned just a few months earlier. They lead schools from their computers, in a school with fewer children, and within the community by connecting online with parents, outreach programs, and assisting organizations. They have become distant leaders, separating their staff and the students who formerly occupied their facilities. The current era is unusual, difficult, and unexpected for leaders in all fields, especially for educational leaders. This estrangement from their pupils is emotionally and psychologically traumatic.

Schools with the most effective leaders groom and develop their own leaders, creating a healthy environment in which all professionals can grow in their abilities and knowledge (Leithwood et al., 2020). This is not inherently impossible from a remote environmental perspective, but it does involve additional effort to maintain meaningful connections with others that sustain relationships and keep things moving. Pragmatically, educational leadership is in turmoil, which calls for chaotic, trial-and-error, stomach-churning leadership in instances of uncertainty, no assurance, and perhaps no resolution. Leadership is a difficult and continual endeavor (Leithwood et al., 2020). Despite everything, educational leaders are adjusting favorably to this crisis in spite of the challenges the pandemic poses. Schools are still in session, as instructors and leadership are preparing for the new school year, and students are still learning.

Globally, COVID-19 represents a massive, audacious, and inevitably irrevocable transformation and test for leaders, including school institutions. Prior to the pandemic, those in charge of educational establishments viewed their leadership tasks from a perspective that had altered little over the decades. This concept of “leadership” has traditionally been connected with positions of authority that are tied to certain job duties, as well as other people's professional assistance. Headteachers ensured that schools successfully and efficiently managed teaching and learning during the pandemic, maintained things under control, were accountable for the performance of schools, and reported to families and governors, including district directorates, in response to the pandemic. This suggests school leaders were inevitably responsible for their decisions, so it is crucial to examine how school leadership managed the pandemic crisis by coordinating with other teachers and stakeholders. Leadership tends to be centered on headteachers during the pandemic, and their leadership response to ensure a smooth transition of change through the pandemic is crucial to the school's development (Harris, 2020).

As remote communication becomes the norm, the organizational landscape of school leaders has transformed radically and irreversibly. In the midst of the present crisis, educational leadership continues to be an important and constructive factor in running school systems. The question is, how does the school's leadership cope with the organizational change caused by the pandemic? In this light, the current review covered how school leadership managed unexpected school transition during the pandemic by adopting path-goal leadership. Specifically, how does the school leadership change commensurate the need to adopt appropriate leadership approaches to coordinate school stuff and other stakeholders to maximize curriculum objectives during the pandemic.

How has the COVID-19 changed school leadership focus? As a result of the COVID-19 catastrophe, leaders had to adapt to rapid adjustments or changes both inside and beyond outside school premises. The plethora of literature on leadership effectiveness of educational transformation mostly centers on those operating in adverse situations. According to Day (2004), educational leaders who successfully lead change accomplish positive results through a genuine commitment and a passionate dedication to their schools' achievement. This is communicated by being humorous, through interpersonal sensitivity, compassion, understanding, and support for the self-esteem of their staff, families, and students (Day, 2004). However, no accessed studies examined the shifting school leadership during a severe national global crisis or has any studies investigated how path goal leadership influence change success.

There is virtually little scientific data on the management of large-scale school lockdown changes. There is pertinent research on crisis-led management in managerialism, like Schmidt and Groeneveld (2021), that concluded that leadership during crisis is typically centered on rapid decision-making mechanisms by the leaders of an organization. Nevertheless, an earlier publication by social psychologists on pandemics (Jetten et al., 2020) suggests that successful leadership amid crisis situations should be centered on building a feeling of connectedness, with leadership collaborating with people and being perceived to stand alongside them' (p. 30). This permits a mutual comprehension of objectives and, consequently, how to proceed jointly.

## Path-Goal Leadership Theory and School Leadership

Path goal theory (PGT) of leading initially emerged during 1970s in the research of Evans (1970), House (1971), House and Dessler (1974), and House and Mitchell (1975), which was refined by House in 1996 (Northouse, 2021). The original concept of the theory included four leadership styles; directive, participative, supportive, and achievement-oriented leadership. According to Northouse (2021), the following is a summary of concept of the leadership styles. Leaders that are directive provide their followers with task directives and guidelines that include their expectations, procedures to execute the tasks, and the duration required to accomplish them. Northouse (2021) stated that performance criteria, norms, and guidelines are specified for followers. The style of leadership is effective inside a high-pressure job setting where subordinates must accomplish difficult objectives and targets. School leaders must maintain cordial and affable interactions with teachers yet maintaining a directive style during the pandemic to achieve cooperation in order to achieve organizational goals. School settings characterized by wholesome and pleasant interactions between teachers and headteachers are productive and positive in the viewpoint of Northouse (2021). These favorable conditions provide more effective directed leadership toward achieving organizational transformation.

Secondly, Northouse (2021) characterizes supportive approach to leadership as both approachable and friendly. The leadership approach addresses the human needs, wellbeing, equality, respect, and acknowledgment of followers particularly when creating aesthetic working conditions. Teachers may provide school leaders with ideas to improve the quality of learning and instruction, or they may engage in significant policy and decision making process as well as the implementation to ensure smooth transformation of school institutions during the pandemic. Participative approach, according to Northouse (2021), enables collaborative decision making in which followers' thoughts and proposals are taking into consideration during policy formulation. To attain all or portions of the desired benefits of participative approach to leadership, school leaders must promote teachers' constant development. In addition, principals must have faith in the abilities of teachers in order for them to accomplish ambitious goals. Participating teachers will able to present everyday problems encountered from the virtual teaching and learning activities, health issues, or family-related situations for redress through headteacher leadership and decision making. Such instances will increase the chances of effective organizational change during the pandemic.

Achievement-oriented leaders communicate their ambitions and goals to their subordinates. They establish goals with high-performing criteria on a regular basis, have faith in their followers' skills, and stimulate continuous performance development (Northouse, 2021). School heads who are directive typically delegate tasks and mostly neglect teachers during administrative decisions in schools. Therefore, a supportive approach educates and reinforces directive leadership to efficiently function. To achieve ambitious aims and goals, supportive leaders create ideal work environments that generate high optimism, job ethics, and dignity. They thus establish the suitability of path goal theory in organizational transformation. School leaders can complement their own leadership styles to maximize the optimal functioning of schools amidst the pandemic crisis. Consequently, an achievement-oriented approach assists educational leaders in establishing aims and targets that encourage teachers to be engaged, enthusiastic, and inspired. Teachers are eligible to participate if they effectively complete their duties based on a set of criteria and in that those teachers are intrinsically compelled to complete assigned tasks on time, which indirectly influences their job performance, hence enhancing the organizational change process.

Incorporating opinions, thoughts, and initiatives, the participative leader combines the knowledge and ingenuity of teachers to find answers to challenges. As such, school leaders must also give teachers some type of redress for errors, negligence, or actions related to the misfeasance of roles. Indisputably, such instances will reinforce an organizational culture ready to accept and successfully transition through the pandemic at the school level with ease through high teacher job performance. Empirical evidence suggest school leadership plays an important role in teacher job performance.

## School Leadership, Teacher Job Performance, and Organizational Change

School leadership is responsible for facilitating and remedying situations within schools from a pragmatic standpoint (Williams-Boyd, 2002; Miller, 2016). In particular, school leaders play a crucial role in enhancing teachers' job performance through their leadership. According to Hamilton (2016), educational leaders have a substantial impact on school achievement. The leadership style of school leaders directly and indirectly affects the productivity of teachers. School leadership brings with it a number of essential roles and responsibilities, including the maintenance of curricular standards; the evaluation of educational methods; the monitoring of student outcomes; the facilitation of teachers; and the creation of an optimistic and achievement-oriented atmosphere in which complex objectives can be attained. Vital components of PGT success (Northouse, 2021) and leadership effectiveness include removing impediments and defining paths for educators to execute their responsibilities. As enablers and problem-solvers, educational leaders provide help in the administrative and academic arenas *via* collections of directives and guidelines to accomplish tasks and attain onerous goals. Effective leadership entails offering a series of directives, including planning steps, such as when and how to execute, motivating subordinates, establishing ambitious goals, and preserving cordial relations. The proclivity of school leaders to follow and carry out the guidelines of path goal theory, as demonstrated by effective teacher support and commitment will empower organizational change in difficult situations such as the COVID-19 pandemic.

Substantial empirical work on PGT efficacy has been published to substantiate the significant effects that the four leadership styles of school leaders provide in terms of teacher employee productivity. Imhangbe et al. (2019) investigated the impact of school leadership on teacher job performance at public senior high schools in Edo, Nigeria and found that a democratic type of leadership had a significant impact on teacher employee productivity. Conversely, Atsebeha (2016) discovered that supportive leader behavior has a reasonably large impact on teacher productivity in Tigray primary schools, Ethiopia. Numerous additional studies on this remarkable phenomenon were done (Somech, 2005; Mwangi, 2013; Machumu and Kaitila, 2014; Okoji, 2016; Wachira et al., 2017).

Teachers' effectiveness and output could decline from poor teacher job productivity and may eventually result in increased personnel turnover, demotivation, and occupational discontent (Aziri, 2011; Saleem et al., 2020), suggesting an unsuccessful organizational transition. Thus, school objectives cannot be met, and student achievement, particularly productivity, would fall in situations of poor teacher productivity. Therefore, school leadership is crucial to school functioning, especially during this pandemic. Effective school leadership will ensure a successful transition for schools through the pandemic. It is necessary to enumerate the effects of PGT school leadership on teacher employee productivity to provide educational leaders, school heads, facilitators, and supervisors with a comprehensive understanding of addressing the leadership issues during a crisis in an attempt to maximize teacher employee productivity and ensure organizational change.

## The Conceptual Framework

Indvik (1985) argues that PGT leadership is applicable to any organizational structure comprised of leaders and followers working toward predetermined objectives. Educational leaders must therefore remove obstacles from the way of their goals in order to assist teachers in achieving their objectives. Every school leader is a problem-solver and goal-attainment facilitator in school organizations (Williams-Boyd, 2002; Miller, 2016). Hamilton (2016) confirmed that educational leaders influence school performance positively. School heads during the pandemic remotely review teaching techniques, monitor student performance, schedule assignments, and assist teachers in fostering an achievement-oriented and encouraging environment conducive to achieving stated objectives. This implies that school leaders eliminate obstacles and clarify the paths for teachers to attain institutional goals (Northouse, 2021), thus fostering organizational change.

Effective leadership includes providing sets of directives, such as planning and carrying out actions, establishing institutional objectives, allocating work, motivating followers, and maintaining and resolving relationships. Despite the pandemic, school leadership assists teachers in both administrative and academic ways as problem-solvers and facilitators by giving instruction manuals and guidelines for goal attainment during the crisis. Saleem et al. (2020) emphasized that a number of studies have proven a significant and direct correlation between the four PGT leadership styles and teacher job performance, suggesting PGT leadership empowers high teacher employee productivity. Therefore, achieving high teacher employee productivity can promote effective organizational change during the pandemic.

**Figure 1:**
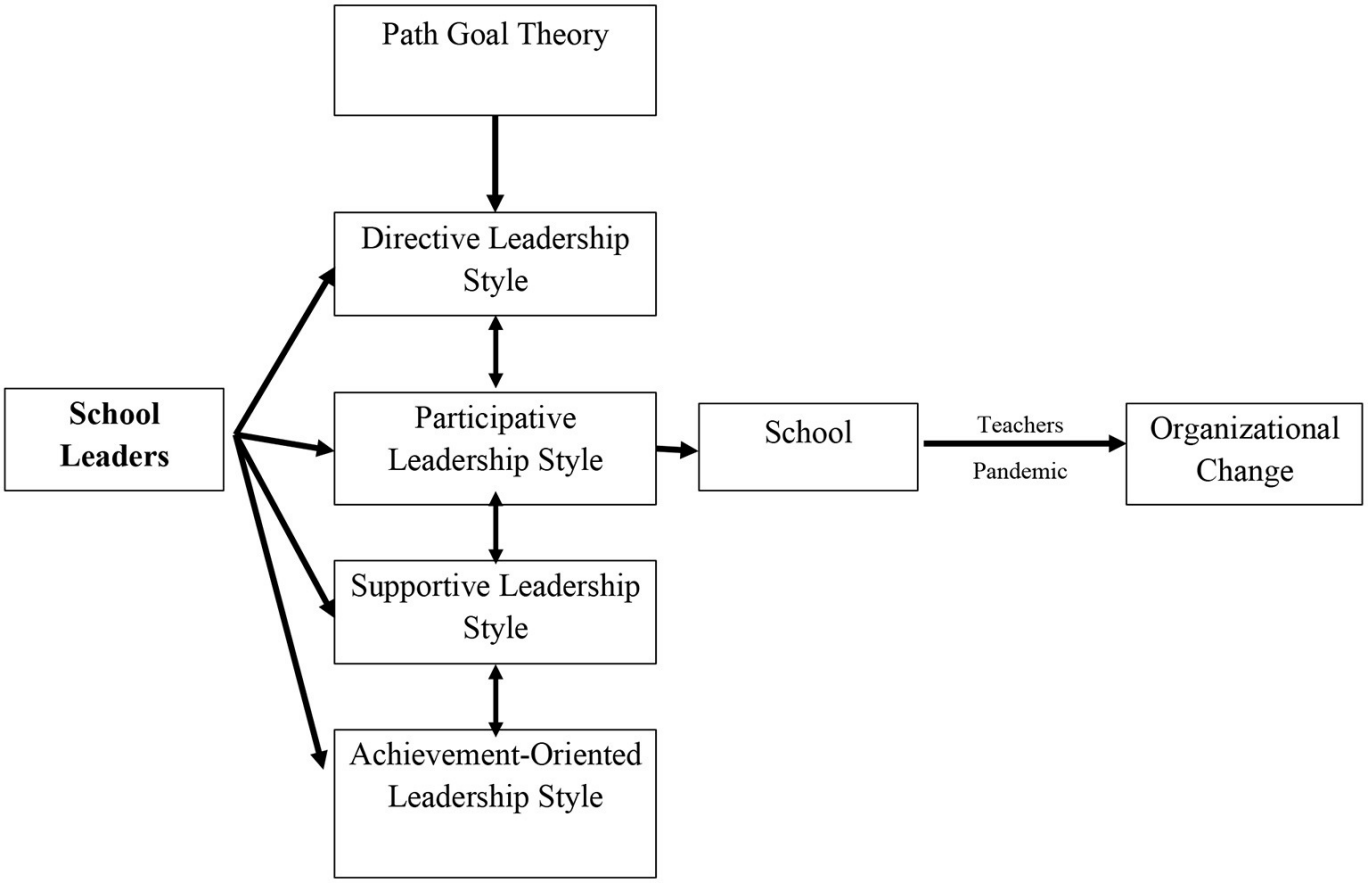
The conceptual framework.

In particular, Atsebeha (2016) and Imhangbe et al. (2019) investigated the association between PGT leadership styles and teacher job performance at the secondary and elementary school levels in Edo, Nigeria and Tigray, Ethiopia, respectively. The first study discovered a substantial correlation between principals' democratic leadership styles and teacher job performance, while the second study proved that leaders' supportive leadership styles significantly impacted teacher job performance. Thus, we confirm the effectiveness of school leadership and teacher productivity in an effort to achieve organizational change. Low teacher job performance could eventually reduce the productive efficiency of teachers, contributing to low employee retention, decreased motivation to complete given tasks, and low job satisfaction (Aziri, 2011). This will ultimately hinder the performance of schools and prevent them from achieving organizational change (Marsh et al., 2009; Khine, 2013; Brown, 2015). It is important to illustrate how PGT leadership styles can foster organizational change through influencing high teacher productivity (see Figure 1).

## Discussion and Conclusion

Path goal theory is a leadership style that is both conceptually and practically challenging. Assumptions are made about how distinct leadership styles interact with the qualities of followers and the workplace environment to influence the motivation of followers especially during crisis. Leaders may use the notion of leadership; directive, participative, supportive, or achievement-centered approaches to help their subordinates do their tasks in an acceptable way. According to the path goal approach, leaders should choose a leadership style that best suits the requirements of their team members and the task they are doing in order to be effective, suggesting the school leadership should to adopt a leadership approach that merits the pandemic situation. As predicted by the theory, it is advisable to use a directed leadership style when dealing with dogmatic and authoritarian subordinates, confusing job requirements, unclear organizational regulations, and difficult tasks. Directed leadership works best in these situations because it gives structure and direction to those who follow (House and Mitchell, 1975). Implacably, the pandemic might have influenced teachers' attention to issues hence, directive leadership in the viewpoint of the researchers could have worked best toward a successful transformational change in the school system caused by the COVID-19 pandemic.

The Path goal theory suggests that leaders should utilize a supportive manner for activities that are regimented, unsatisfactory, or difficult. When people are doing repetitive and uninteresting work, the supportive leadership style fills the void. Leaders that provide a feeling of personal touch to their subordinates as they do routine, mechanical tasks are known as “supportive leaders.” When a task is uncertain, participatory leadership is the best option because it helps leaders remove obstacles in the way of goals (House and Mitchell, 1975; Northouse, 2021). Significantly, the pandemic brought forth repetitive practices such as everyday hygiene activities, classroom practices, and social distancing activities which are supposed to be observed every time. Supportive and participative leadership styles would be ideal to reinforce such practices as teachers, students, and parents may find them redundant. To add to the beneficial influence of participative leadership, this kind of follower reacts positively to being included in decision-making and in the structure of their job. For this reason, achievement-oriented leadership is most successful in situations in which followers must accomplish tasks that are both clear and ambiguous, according to the path–goal theory; leaders that raise the bar for their subordinates and encourage them to strive for excellence do wonders for their followers' self-esteem and motivation. Achievement-oriented leadership encourages followers to believe that their hard work will pay off (Saleem et al., 2020, 2021; Northouse, 2021). But achievement-oriented leadership doesn't seem to have anything to do with what people expect from their work efforts when the job is clearer and more well-defined.

School leadership in light of PGT would set clear goals such as “we should achieve zero infections for this week”. To achieve such a goal, teachers, students, and parents would be educated about the goal, and supported with the discourse required to achieve it, indicating PGT is influential in promoting high productivity. Additionally, teachers, parents, and students will be included in the decision making process which will give them adequate knowledge of the goals hence work toward achieving it.

Path–goal theory is simple to understand from a logical standpoint. A good leader must pay attention to the requirements of his or her subordinates especially, during crisis like the COVID-19 pandemic. The leader's role is to assist the followers in identifying and pursuing their own objectives. A leader's role is to assist those under his or her care in overcoming setbacks. This might mean eliminating the impediment, or it could mean helping the follower get past it. As a leader, it's your job to guide, teach, and coach your subordinates as they try to reach their goals. Future studies should test the PGT leadership style that works best during crisis. The leadership theory should also be test among followers of different abilities and experiences in different contexts to compare its effectiveness.

## Author Contributions

All authors listed have made a substantial, direct, and intellectual contribution to the work and approved it for publication.

## Conflict of Interest

The authors declare that the research was conducted in the absence of any commercial or financial relationships that could be construed as a potential conflict of interest.

## Publisher's Note

All claims expressed in this article are solely those of the authors and do not necessarily represent those of their affiliated organizations, or those of the publisher, the editors and the reviewers. Any product that may be evaluated in this article, or claim that may be made by its manufacturer, is not guaranteed or endorsed by the publisher.
